# Sleep Disturbance Induces Increased Cholesterol Level by NR1D1 Mediated CYP7A1 Inhibition

**DOI:** 10.3389/fgene.2020.610496

**Published:** 2020-12-23

**Authors:** Chen Xing, Xin Huang, Yifan Zhang, Chongchong Zhang, Wei Wang, Lin Wu, Mengnan Ding, Min Zhang, Lun Song

**Affiliations:** ^1^ Institute of Military Cognitive and Brain Sciences, Academy of Military Medical Sciences, Beijing, China; ^2^ School of Basic Medicine, Henan University, Kaifeng, China; ^3^ School of Pharmacy, Jiamusi University, Jiamusi, China

**Keywords:** CYP7A1, circadian clock, cholesterol, sleep disturbance, NR1D1

## Abstract

Disturbed sleep is closely associated with an increased risk of metabolic diseases. However, the underlying mechanisms of circadian clock genes linking sleep and lipid profile abnormalities have not been fully elucidated. This study aimed to explore the important role of the circadian clock in regulating impaired cholesterol metabolism at an early stage of sleep deprivation (SD). Sleep disturbance was conducted using an SD instrument. Our results showed that SD increased the serum cholesterol levels. Concentrations of serum leptin and resistin were much lower after SD, but other metabolic hormone concentrations (adiponectin, glucagon, insulin, thyroxine, norepinephrine, and epinephrine) were unchanged before and after SD. Warning signs of cardiovascular diseases [decreased high density lipoprotein (HDL)-cholesterol and increased corticosterone and 8-hydroxyguanosine levels] and hepatic cholestasis (elevated total bile acids and bilirubin levels) were observed after SD. Cholesterol accumulation was also observed in the liver after SD. The expression levels of HMGCR, the critical enzyme for cholesterol synthesis, remained unchanged in the liver. However, the expression levels of liver CYP7A1, the enzyme responsible for the conversion of cholesterol into bile acids, significantly reduced after SD. Furthermore, expression of NR1D1, a circadian oscillator and transcriptional regulator of CYP7A1, strikingly decreased after SD. Moreover, NR1D1 deficiency decreased liver CYP7A1 levels, and SD could exacerbate the reduction of CYP7A1 expression in *NR1D1^−/−^* mouse livers. Additionally, NR1D1 deficiency could further increase serum cholesterol levels under SD. These results suggest that sleep disturbance can induce increased serum cholesterol levels and liver cholesterol accumulation by NR1D1 mediated CYP7A1 inhibition.

## Introduction

Maintenance of sleep homoeostasis regulated by the homoeostatic and circadian process plays important roles in the balance of psychological and physical health (Saper et al., 2005; Kon et al., 2017). However, short sleep duration or disturbed sleep due to shift work, jet lag, and insomnia is becoming a widely social and medical problem in modern society (Kecklund and Axelsson, 2016; Bertisch et al., 2018). Numerous epidemiological studies have suggested that adverse effects of sleep loss are closely associated with increased risk of obesity, type 2 diabetes, and cardiovascular diseases (CVDs; Reutrakul and Van Cauter, 2018; Liu and Chen, 2019). The association of metabolic disorders with sleep loss caused by total sleep deprivation (SD), chronic sleep restriction, or sleep fragmentation has gained attention (Killick et al., 2012; Nedeltcheva and Scheer, 2014). It has been suggested that the development of metabolic disorders or CVDs may be associated with long term or immediate effects of sleep loss or disruption. Thus, it is important to explore the potential mechanism underlying this association.

The circadian clock is regarded as the endogenous timekeeper of many day to day biological and physiological processes, including the sleep-wake cycle and cyclic metabolic changes (Panda, 2016; Kon et al., 2017). The circadian clock is located in the suprachiasmatic nucleus and peripheral tissues, and it synchronizes physiology and behavior throughout the 24-h day-night cycle (Takahashi, 2017). It has been suggested by some experimental and epidemiological reports that misalignment or disruption of the circadian clock can increase the risk of metabolic diseases. For example, the genetic model of clock mutant mice is susceptible to obesity, hyperlipidaemia, hyperglycaemia, and hepatic steatosis (Turek et al., 2005). Additionally, knockout of clock genes, such as aryl hydrocarbon receptor nuclear translocator-like (ARNTL), PERIOD (PER2), and cryptochrome (CRY1/CRY2), increased their susceptibility to obesity and metabolic disorders (Yang et al., 2009; Marcheva et al., 2010; Hemmeryckx et al., 2011; Shimba et al., 2011; Barclay et al., 2013). Polymorphisms of human clock genes, such as ARNTL, circadian locomotor output cycles kaput (CLOCK), CRY2, and nuclear receptor subfamily 1, group D, member 1 (NR1D1), have been linked to obesity or some other features of metabolic syndromes (Woon et al., 2007; Sookoian et al., 2008; Ruano et al., 2014; Machicao et al., 2016). Clinical studies on chronic circadian misalignment due to shift work reported an increased incidence of diabetes, obesity, and CVDs in the study participants (Khan et al., 2018; Torquati et al., 2018; Chellappa et al., 2019). Thus, disruption of the circadian clock may contribute to the development of obesity and metabolic diseases.

Emerging evidence has revealed that total sleep deprivation or sleep restriction influences concentrations of lipid profiles in plasma (e.g., triglyceride, cholesterol, and lipoproteins), suggesting the role of sleep in regulating lipid metabolism (Chua et al., 2015; Brianza-Padilla et al., 2016). Additionally, it has been reported that insufficient or disturbed sleep can lead to an impairment of rhythmic transcriptomes of central and peripheral tissues, including the genes associated with circadian rhythm and metabolism (Archer and Oster, 2015). However, the underlying mechanisms of circadian clock genes linked to sleep duration and lipid profile abnormalities have not been fully understood.

Therefore, this study aimed to explore the role of the circadian clock in regulating impaired lipid metabolism under disturbed sleep conditions. Sleep disturbance measurement was conducted by a sleep deprivation instrument. Total cholesterol, triglycerides, free fatty acid, some risk factors for hepatic cholestasis, and CVDs were detected first. Pathological staining of the liver, cholesterol synthesis, and conversion of cholesterol to bile acids were also assessed during the study. Finally, the NR1D1 function in liver cholesterol accumulation induced by sleep disturbance was analyzed.

## Materials and Methods

### Animals and Sleep Deprivation

The male Sprague-Dawley rats and C57BL/6J mice aged at 6–8 weeks were purchased from Charles River Laboratory Animal Technology Co. (Beijing, China). The NR1D1^−/−^ mice were purchased from Cyagen Technology Co. (Guangzhou, China). The rats and mice were bred in animal facilities under specific pathogen-free conditions with a 12:12 h light-dark cycle (light on at 06:00 AM and light off at 18:00 PM). Morning 06:00 (zeitgeber time) was considered as the ZT 0. Food and water were available ad libitum. The rats and mice were randomly divided into experimental and control groups. Animals in the experimental group were subjected to the sleep deprivation instrument (DB036, BEIJING ZHISHU DUOBAO BIOLOGICAL TECHNOLOGY CO., LTD), which used a randomly moving platform to keep animals awake. The platform provides separate cages to allow animals free access to food and water. Parameters for the moving platform can be set by the touch screen of the controller. Additionally, the parameters can be changed by programming to reduce the adaptation of sleep deprived animals to the environment. After the parameters are set, sleep deprivation can be carried out in rats or mice without human intervention. Sleep deprivation was carried out at ZT 0, and animals were sacrificed after SD for 72 h. Then fresh blood samples and liver tissues were obtained for further analysis. The instrument does not need to train animals and can limit sleep in a mild way, which could greatly reduce the damage caused by the traditional water environment sleep deprivation method. Care, use, and treatment of rats and mice in this study were in strict agreement with international guidelines for the care and use of laboratory animals. This study was approved by the Animal Ethics Committee of the Beijing Institute of Basic Medical Sciences.

### Serum Lipids and Metabolic Related Hormones Assay

Fresh blood samples were obtained and then centrifuged at 2000 *g* at 4°C for 20 min. The serum was transferred into a new tube and stored at −80°C until use. The serum levels of cholesterol (CEB701Ge), triglycerides (CEB687Ge), leptin (SEA084Ra), resistin (RETN) (SEA847Ra), adiponectin (ADP) (SEA605Ra), thyroxine (CEA452Ge), epinephrine (CEA858Ge), norepinephrine (CEA907Ge), insulin (CEA448Ra), glucagon (CEB266Ra), corticosterone (CEA540Ge), 8-hydroxyguanosine (8-OHdG) (CEA660Ge), alkaline phosphatase (ALP) (SEB472Ra), and bilirubin (CEK522Ge) were measured using the corresponding ELISA kits (CLOUD-CLONE CORP, China). Concentration of high density lipoprotein (HDL)-cholesterol (HDL-C; ab65390, Abcam), free fatty acid (ab65341, Abcam), and total bile acids (ab239702, Abcam) were analyzed with the corresponding assay kits. All the assay procedures were performed according to the protocols provided by the manufacturer.

### Hepatic Lipids Assay

Rat hepatic lipids were determined by the method as described previously (Becares et al., 2019). Briefly, liver tissues were homogenized in 10 mM Tris buffer with 250 mM sucrose and 2 mM EDTA. Hepatic lipids in the homogenates were extracted with chloroform:methanol solution (1:1) by shaking for 3–4 h at room temperature. The homogenates were vortexed and centrifuged at 15000 *g* to obtain the organic phase. The organic phase could be dried with nitrogen and dissolved in 0.01 M phosphate buffer saline (PBS, with 1% Triton X-100). Then, further analyses of cholesterol (CEB701Ge), HDL (SEB006Ra), triglycerides (CEB687Ge), and very low density lipoprotein (VLDL; E-EL-R1203c, Elabscience) were performed according to the protocols provided by the manufacturer.

### Oil Red O Staining

The rats were anesthetized and then transcardially perfused with normal saline, 4% paraformaldehyde in 0.01 M PBS. Liver tissues were dissected out, fixed in 4% PFA in 0.01 M PBS at 4°C overnight, transferred into 30% (w/v) sucrose in PBS for 24 h, embedded in tissue freezing medium (JUNG, Leica Biosystems), and stored at −80°C. Livers samples were cryosectioned into 10 μm thick sections and mounted on glass slides. The sections were stained with 0.3% Oil red O solution, counterstained with hematoxylin, rinsed under running tap water, and coverslipped with aqueous mountant.

### Hematoxylin-Eosin Staining

Hematoxylin-eosin (HE) staining was conducted according to routine protocols. Liver tissues of six rats from each group were fixed in 10% neutral buffered formalin for 48 h, and then embedded in paraffin and sectioned into 3–5 μm thick sections. After deparaffinization and rehydration, the sections were stained with hematoxylin, dipped in 1% acid ethanol, and rinsed in distilled water. Then, the sections were stained with eosin solution and followed by with graded alcohol dehydration. The mounted slides were photographed by an Olympus BX53 fluorescence microscope (Tokyo, Japan).

### Western Blot Analysis

Fresh liver samples were harvested, washed with cold PBS, and lysed with ice-cold lysis buffer supplemented with protease inhibitors. Cell lysate was run on 10% SDS-PAGE, transferred to a polyvinylidene fluoride membrane (PVDF; ISEQ00010, Millipore), blocked with 5% skim milk, and incubated with primary antibodies at 4°C overnight. The primary antibodies against 3-hydroxy-3-methylglutaryl-CoA reductase (HMGCR; sc-271595, Santa Cruz Biotechnology), cytochrome P450 family 7 subfamily A member 1 (CYP7A1; sc-518007, Santa Cruz Biotechnology), NR1D1 (sc-323,915, Santa Cruz Biotechnology), and β-actin (sc-8432, Santa Cruz Biotechnology) were used. Then, the membrane was incubated with the corresponding horseradish peroxidase (HRP)-conjugated secondary antibodies for 1 h at room temperature. After extensive washing three times, the bands were detected using an ECL detection system (4600SF, Tanon Science & Technology Co., Ltd).

### RT-PCR Analysis

Total RNA was isolated from fresh liver tissues using Trizol reagent (T9424, Sigma Aldrich) according to the manufacturer’s instructions. After concentration determination, the cDNA products were obtained using All-in-One cDNA Synthesis SuperMix (B24403, Biotool) for reverse transcription. The amplification of target genes was performed using the standard protocol by Bio-Rad T100TM Thermal Cycler (1861096, Bio-Rad). The rat primers used were as follows: HMGCR-F: 5'-gcctccattgagtccggagg-3', HMGCR-R: 5'-gcaccgggttatcgtgagga-3'; CYP7A1-F: 5'-gaccaagtctttccggcac-3', CYP7A1-R: 5'-cagagaatagcgaggtgcgt-3'; FXR-F: 5'-aggaagtgcagagagatggga-3', FXR-R: 5'-agcttggtcgtggaggtcac-3'; RXRa-F: 5'-cgctcctcaggcaaacacta-3', RXRa-R: 5'-ggaggatgccgtctttcaca-3'; SHP-F: 5'-aggctcactgggcattgtgt-3', SHP-R 5'-acatctccgatgacagggcg-3'; HNF4a-F: 5'-aaaaccctcgccgacatgga-3', HNF4a-R: 5'-ctgacacccaggctgttgga-3'; C/EBPa-R: 5'-tggagggttcttagcccctt-3', C/EBPa-F: 5'ggctggcgacatacagtaca-3'; NR1D1-F: 5'-tacaagtggccatggaagaca-3', NR1D1-R: 5'-ttggccaagttcatggcgttc-3'; E4BP4-F: 5'-ctgatgcagctgagaaaaatgc-3', E4BP4-R: 5'-gggagagcagctcagctttta-3'; R-GAPDH-R: 5'-cagttccagcctcgtctcat-3', GAPDH-F: 5'-actgtgccgttgaacttgcc-3'. The mouse primers used were as follows: NR1D1-F: 5'-acgaccctggactccaataa-3', NR1D1-R: 5'-ccattggagctgtcactgtaga-3'; β-actin-F: 5'-ttgccctagacttcgagcaa-3', β-actin-R: 5'-caggaaggaaggctggaaga-3'. Gene relative expression was normalized to the mRNA levels of β-actin.

### Statistical Analysis

Statistical analysis was performed using GraphPad Prism 5 (GraphPad Software Inc., San Diego, CA, United States). Data are presented as the mean ± SE and analyzed using the two-tailed, nonpaired Student’s *t*-test for comparison of two groups. The significant difference was set at *p* < 0.05.

## Results

### SD Induced Increased Cholesterol Concentration in Serum Lipid Profiles

**Figure 1:**
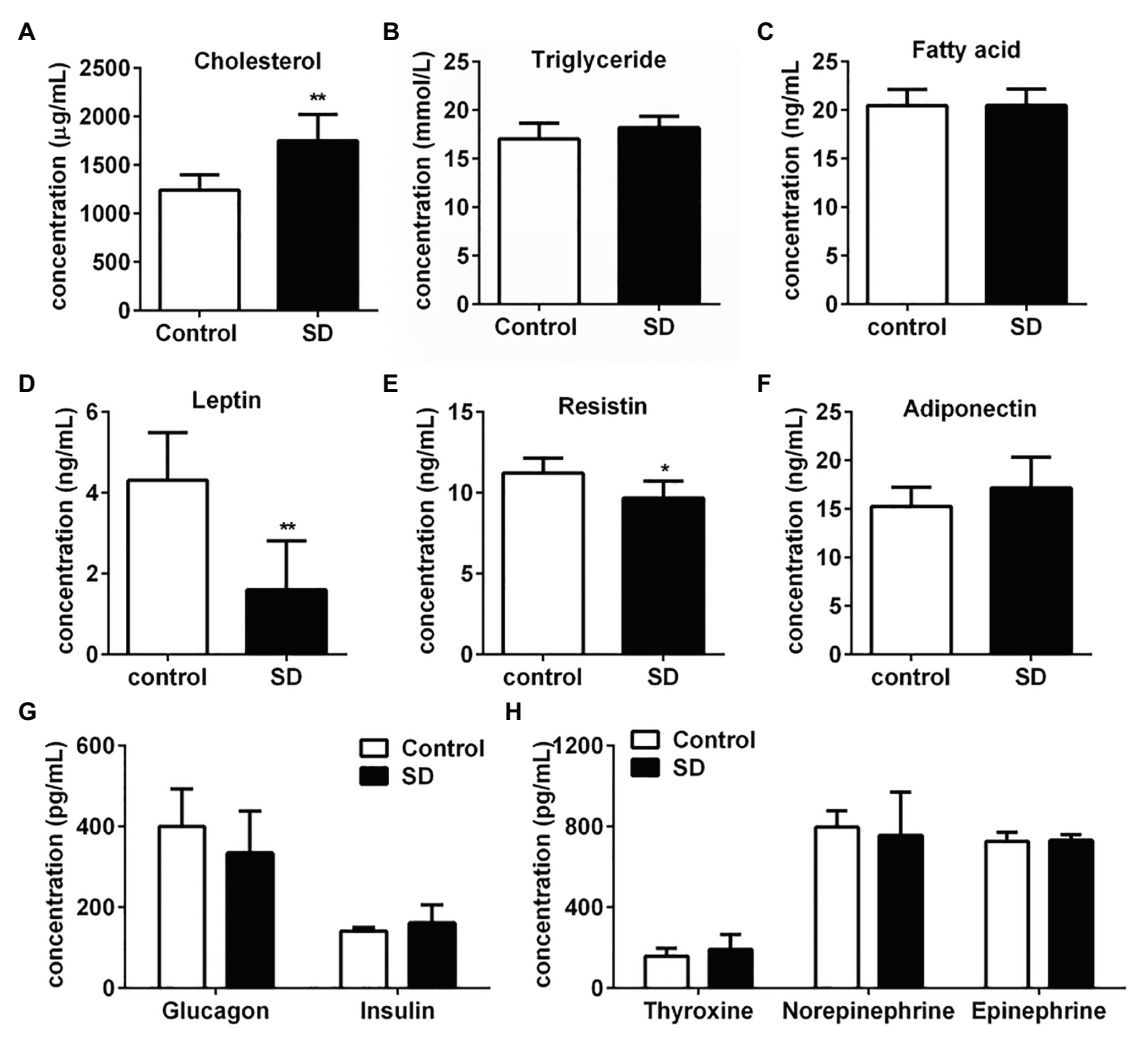
Sleep deprivation (SD) induced increased cholesterol concentration in serum lipid profiles. **(A)** Cholesterol, **(B)** triglyceride, and **(C)** free fatty acids concentrations in rat serums were analyzed under disturbed sleep condition for 72 h. Metabolism related hormones including **(D)** leptin, **(E)** resistin (RETN), **(F)** adiponectin (ADP), **(G)** glucagon and insulin, **(H)** thyroxine, norepinephrine, and epinephrine concentration in rat serums were detected. ^**^p < 0.01; ^*^p < 0.05.

Rat serum samples were analyzed to evaluate the metabolic effects induced by SD. SD for 72 h induced significant increasing of cholesterol level, while triglycerides and free fatty acids levels kept unchanged compared with the control group (Figures 1A–C). Hypercholesterolemia is a metabolic disorder characterized by increased cholesterol levels in circulation. The above results indicated that the tendency of hypercholesterolemia occurrence was much earlier than hypertriglyceridemia induced by SD. Some hormones secreted during the sleep-wake cycle contribute to the metabolism and energy balance of the body. Therefore, concentrations of some hormones involved in metabolism regulation and appetite control were determined. The results showed that concentration of serum leptin and resistin in the SD group were much lower than that in the control group (Figures 1D,E). However, serum levels of some other hormones, including adiponectin, glucagon, insulin, thyroxine, norepinephrine, and epinephrine, slightly differed in both groups (Figures 1F–H). The above results suggested that high cholesterol level in serum lipid profiles was induced by disturbed sleep stress.

### SD Induced the Warning Signs of an Increased Risk of Cardiovascular and Hepatic Diseases

**Figure 2:**
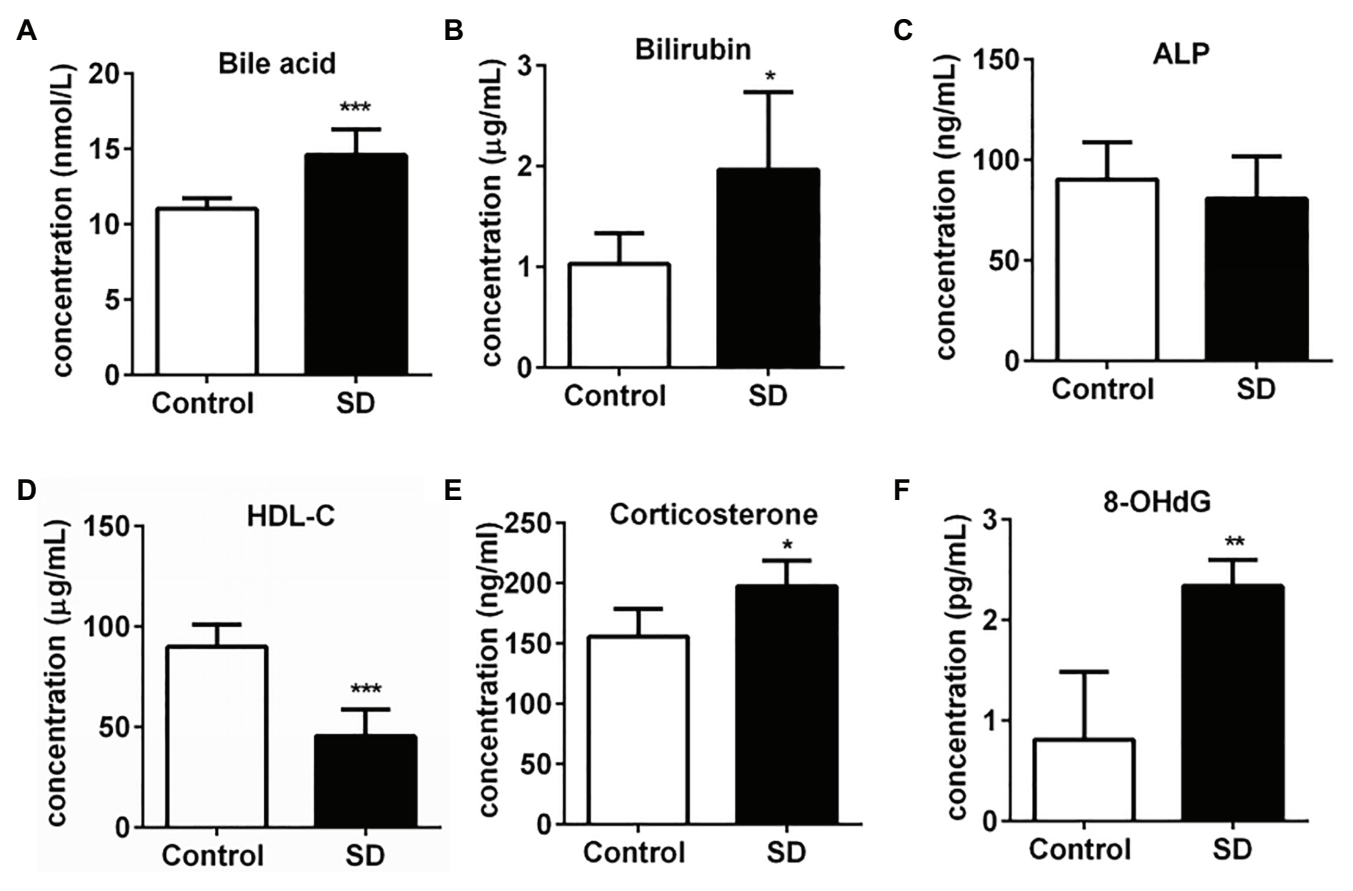
Sleep deprivation induced the warning signs of an increased risk of cardiovascular and hepatic diseases. **(A)** Total bile acids, **(B)** bilirubin, and **(C)** alkaline phosphatase (ALP) concentrations in rat serums increased after sleep disturbance for 72 h. The decreased concentration of **(D)** high density lipoprotein (HDL)-cholesterol (HDL-C) and increased concentration of **(E)** corticosterone and **(F)** 8-hydroxyguanosine (8-OHdG) in rat serums were induced by SD. ^***^p < 0.001; ^**^p < 0.01; ^*^p < 0.05.

A high level of serum cholesterol or hypercholesterolemia is closely associated with increased risk of CVDs and hepatic diseases due to the induction of various stress pressures. In the experimental group, the total serum bile acid and bilirubin levels significantly increased after SD, but alkaline phosphatase (ALP) concentration remained unchanged in serum (Figures 2A–C). The results suggest the risk of hepatic cholestasis caused by SD. Simultaneously, the level of some risk factors for CVDs such as corticosterone and 8-OHdG in serum were found to be notably increased, while the concentration of protective component predicting cardiovascular risk HDL-C was markedly decreased in the SD group (Figures 2D–F). The above results together suggested the warning signs of future hepatic diseases and CVDs.

### SD Induced Accumulation of Cholesterol in the Liver

**Figure 3:**
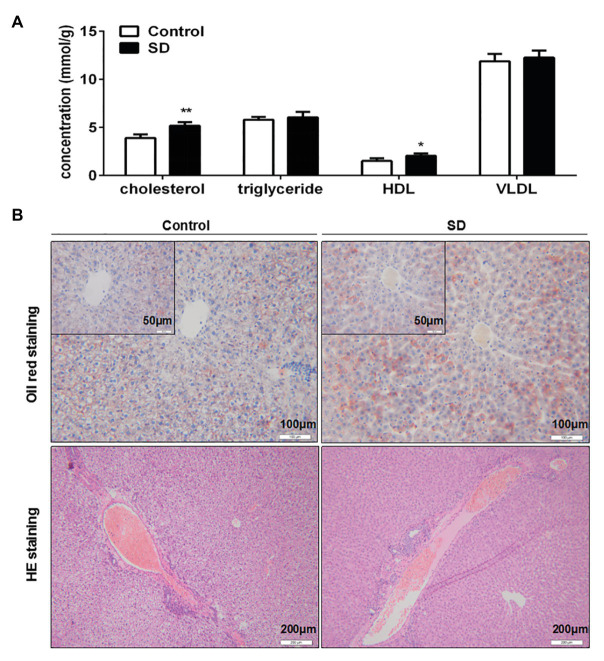
Sleep deprivation induced accumulation of cholesterol in liver. **(A)** The level of cholesterol, triglyceride, HDL, and very low density lipoprotein (VLDL) in rat liver lipid profiles were analyzed. **(B)** Pathological staining of rat livers was assessed by oil red O staining and hematoxylin-eosin (HE) staining. ^**^p < 0.01; ^*^p < 0.05.

Synthesis and degradation of cholesterol mainly occurs in the liver. It was found that cholesterol and HDL levels in rat liver were significantly increased after SD (Figure 3A). Meanwhile, triglycerides and VLDL levels in liver remained unchanged by SD (Figure 3A). Moreover, no obvious lipid droplets were observed in the pathological results of oil red O staining, but the coloring degree of staining was seemingly slightly enhanced after SD (Figure 3B), and there was no distinct increasing of inflammatory cells invasion by HE staining after SD (Figure 3B). The above results indicate that sleep disturbance induced an abnormal cholesterol metabolism in the liver before the occurrence of other pathological changes.

### Bile Acid Synthesis Was More Vulnerable to SD Than Cholesterol Synthesis

**Figure 4:**
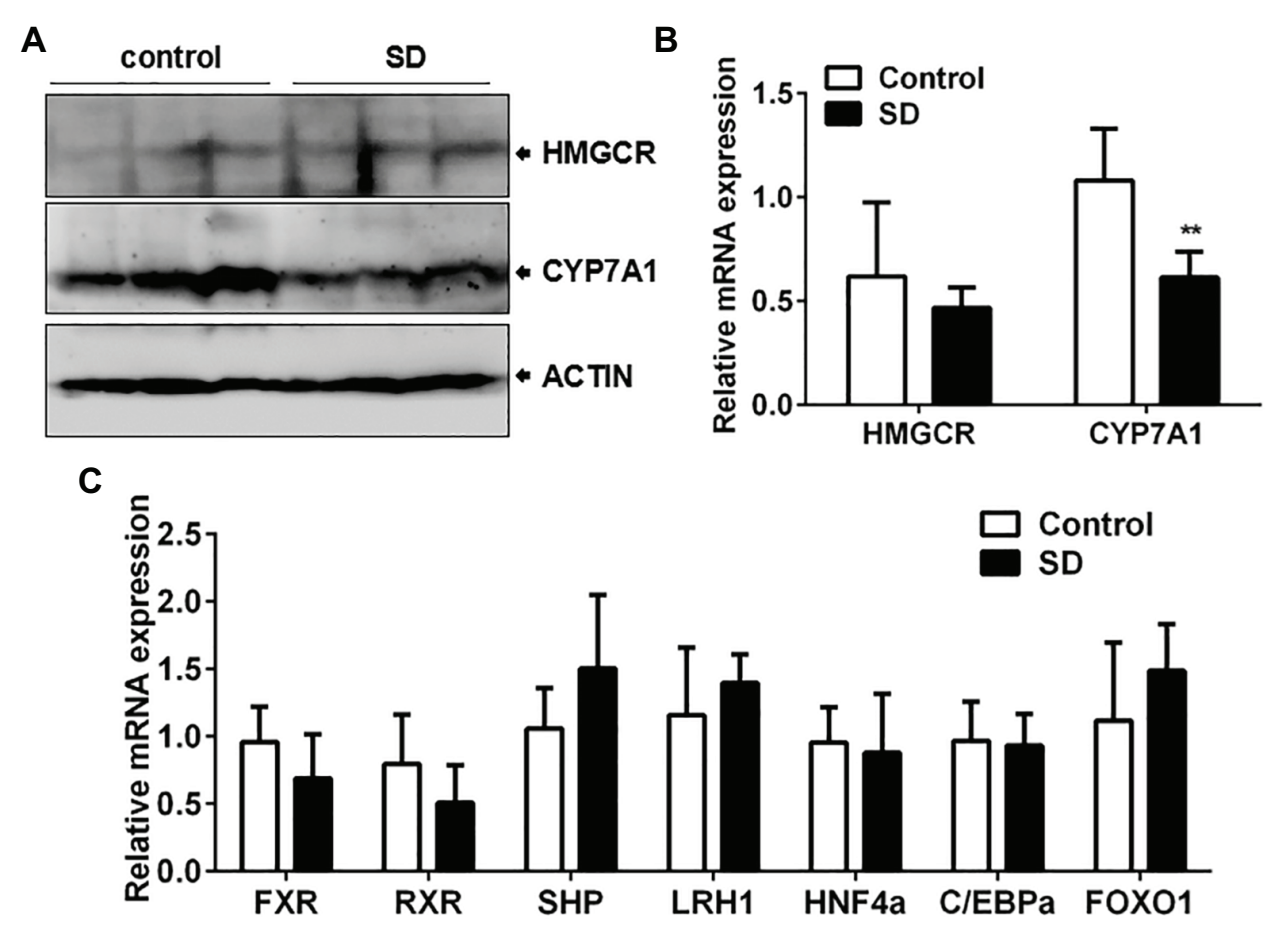
Conversion of cholesterol to bile acids was affected by SD. **(A)** The protein and **(B)** relative mRNA expression of cytochrome P450 family 7 subfamily A member 1 (CYP7A1) and 3-hydroxy-3-methylglutaryl-CoA reductase (HMGCR) in rat livers. **(C)** Expression of some reported bile acid synthesis regulators including farnesoid-X-receptor (FXR), retinoid X receptor alpha (RXRa), liver receptor homolog 1 (LRH1), hepatocyte nuclear receptor 4 alpha (HNF4a), CCAAT enhancer binding protein α (C/EBPa), and forkhead box O1 (FOXO1) were not strikingly affected by SD. ^**^p < 0.01.

To determine the reason behind high serum cholesterol levels, the synthesis and degradation of cholesterol in the liver were analyzed. Cholesterol synthesis and its conversion to bile acids are necessary to ensure cholesterol homoeostasis. The two rate-controlling enzymes involved in cholesterol and bile acid synthesis are HMGCR and CYP7A1, respectively. The expression of CYP7A1 mRNA and protein in rat livers was downregulated but not HMGCR expression after SD (Figures 4A,B). In addition, liver CYP8B1 expression, a downstream enzyme of CYP7A1, was also weakened under the SD condition. However, expression of CYP27A1, a critical enzyme in the alternative pathway of bile acid synthesis, remained unchanged after SD (Supplementary Figure 1). The results indicate that bile acid synthesis was more vulnerable to SD than cholesterol synthesis. Additionally, bile acid synthesis regulators were analyzed. The results showed that expression levels of farnesoid-X-receptor (FXR), retinoid X receptor alpha (RXRa), liver receptor homolog 1 (LRH1), hepatocyte nuclear receptor 4 alpha (HNF4a), CCAAT enhancer binding protein α (C/EBPa), and forkhead box O1 (FOXO1) at transcription level were not strikingly affected by SD (Figure 4C). The above results suggest that bile acid synthesis was partially inhibited under disturbed sleep conditions, which indicates that the conversion of cholesterol to bile acids is more susceptible to SD than cholesterol synthesis.

### Correlation of NR1D1 With Bile Acid Synthesis Under Disturbed Sleep Conditions

**Figure 5:**
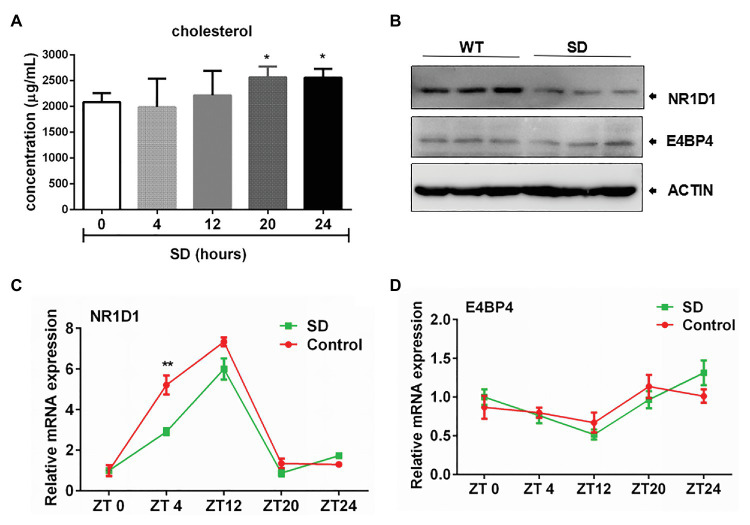
Correlation of nuclear receptor subfamily 1, group D, member 1 (NR1D1) with bile acid synthesis under sleep disturbance condition. **(A)** Increasing of cholesterol level in rat serum induced by acute SD in 24 h. **(B)** NR1D1 and E4, promoter binding protein 4 (E4BP4) expression in rat liver after SD for 72 h. Oscillation expressions of **(C)** NR1D1 and **(D)** E4BP4 in rat liver under acute SD stress. ^**^p < 0.01; ^*^p < 0.05.

Hepatic cholesterol and bile acid synthesis are controlled by the circadian clock. We found that SD for 24 h could induce obvious increasing of cholesterol level in rat serum (Figure 5A). The rapid increase in cholesterol levels induced by an acute SD of 24 h suggests the potential role of circadian clock in regulating cholesterol metabolism. The rapid increase in cholesterol level induced by an acute SD in 24 h suggests the potential role of the circadian clock in regulating cholesterol metabolism. NR1D1 and E4 promoter binding protein 4 (E4BP4) have been reported to participate in regulation of CYP7A1 expression. It was found that NR1D1 expression was downregulated after SD for 72 h but not E4BP4 expression, which remained unchanged (Figure 5B). The expression of CYP7A1 in liver displayed diurnal oscillation variation (data not shown). Further analyses demonstrated that amplitudes of NR1D1 expression were partially decreased by acute SD of 24 h. NR1D1 expression could exhibit a rapid response to SD at ZT4, but the pattern of E4BP4 expression remained unchanged by an acute SD of 24 h (Figures 5C,D). The above results suggested the role of NR1D1 in regulating CYP7A1 expression under disturbed sleep conditions.

### High Cholesterol Level Induced by SD Correlated With Downregulation of CYP7A1 Expression by NR1D1

**Figure 6:**
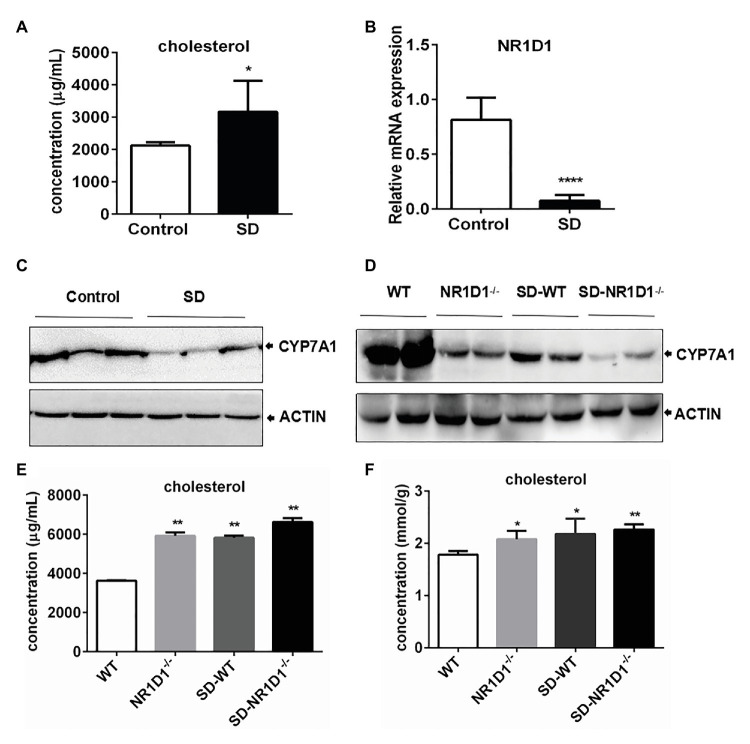
High level cholesterol induced by SD correlated with downregulation of CYP7A1 expression by NR1D1. **(A)** Increasing of cholesterol concentration in mice serums induced by SD. **(B)** Relative mRNA expression of NR1D1 in mice livers after SD. **(C)** Inhibition of CYP7A1 expression in mice livers after SD. **(D)** Reduction of CYP7A1 expression in sleep disturbed NR1D1^−/−^ mice. **(E)** Serum cholesterol level in sleep disturbed NR1D1^−/−^ mice. **(F)** liver cholesterol level in sleep disturbed NR1D1^−/−^ mice. ^****^p < 0.0001; ^**^p < 0.01; ^*^p < 0.05.

In order to figure out the potential role of NR1D1 in CYP7A1 expression, we compared the responses of wild type (WT) and NR1D1^−/−^ mice after SD. Similarly, SD could induce an obvious increase in serum cholesterol levels along with downregulation of NR1D1 and CYP7A1 expression in the livers of WT mice (Figures 6A–C). Furthermore, NR1D1 deficiency downregulated the expression levels of CYP7A1, and sleep disturbance could exacerbate the decreased CYP7A1 expression (Figure 6D). Additionally, NR1D1 deficiency could also further increase serum cholesterol level and the tendency of cholesterol accumulation in the liver under sleep disturbance conditions (Figures 6E,F). The above data indicated that downregulation of NR1D1 expression by the SD-mediated transcriptional inhibition of CYP7A1, leading to the block of cholesterol degradation and high level cholesterol accumulation in serum.

## Discussion

Accumulating evidence from the epidemiologic and laboratory studies have suggested the role of reduced or disturbed sleep in the metabolic dysfunction, leading to an increased risk of type 2 diabetes, obesity, or weight gain. Considering the early metabolic alterations caused by sleep disturbance, our results showed that a high cholesterol level in serum lipid profiles was rapidly induced by sleep deprivation exposure (24 and 72 h) in both rat and mice. Therefore, influences on cholesterol homeostasis regulation should be the early metabolic related events urged by sleep disturbance. Long term and uncontrolled exposure to hypercholesterolemia can lead to a slow and progressive occurrence of cardiovascular events (Jeong et al., 2018). In our study, sleep disturbance induced some early signs of future CVDs. Plasma HDL-C was decreased under disturbed sleep stress. The cholesterol contained in HDL is verified to be inversely associated with the risk of coronary heart disease and a predictor of cardiovascular risk for its central role in reverse cholesterol transport (Rader and Hovingh, 2014; Ouimet et al., 2019). Besides, HDL has also been proved to exert inhibitory roles on oxidative pathways in CVDs (Kontush and Chapman, 2010; Brites et al., 2017). High levels of 8-OHdG, a biomarker of oxidative stress, have been observed in patients with severe atherosclerotic lesions and hypertension (Martinet et al., 2002; Xiang et al., 2011; Rosello-Lleti et al., 2012). Therefore, the elevated 8-OHdG level observed in this study reminds the future rising oxidative damage induced by SD. Accordingly, the above risk factors together suggest that hypercholesterolemia induced by SD might predict the warning sign of CVDs by affecting multiple blood parameters related to the cardiovascular system.

High plasma cholesterol can contribute to the development of liver disorders, such as non-alcoholic fatty liver disease (NAFLD) and non-alcoholic steatosis hepatitis (NASH) (Kerr and Davidson, 2012; Ioannou, 2016). Our results found the elevated level of bilirubin, bile acids, and cholesterol in serum under disturbed sleep stress. Bilirubin, bile acids, and cholesterol are important components of bile, and impairment in their formation or excretion can lead to cholestasis (Paumgartner, 2010). Particularly, the accumulation of bile acids in the serum and hepatocytes suggests the increased risk of cholestasis occurrence at early stage of liver diseases under disturbed sleep stress. Cholestasis is usually caused by alterations of hepatobiliary transport systems in hereditary cholestasis or acquired cholestatic liver diseases (Lee and Boyer, 2000; Sticova et al., 2018). Apart from mutations in transporter genes, pro-inflammatory cytokines, hormones, and drugs are major pathogenic factors for acquired forms of cholestasis (Stieger et al., 2000; Zollner and Trauner, 2006). Thus, the results of our study indicate that short sleep duration or sleep disturbance also should be a risk factor for cholestatic liver diseases.

Various epidemiological studies have reported that short sleep duration is associated with an elevated weight and body mass index (BMI; Kobayashi et al., 2012; Bayon et al., 2014). Being overweight is associated with greater energy intake than expenditure. Though some reported hormones involved in regulation of lipid and glucose metabolism and triglycerides levels kept unchanged, leptin concentration was found to be decreased under disturbed sleep stress for 72 h in this study. Leptin plays a major role in energy homoeostasis through appetite control and food intake limitation. It has been reported that insufficient sleep promotes dietary intake and contributes to the development of obesity and metabolic alteration (Prinz, 2004; Taheri et al., 2004). Thus, the decline in serum leptin levels induced by SD is in agreement with earlier reports on the links between short sleep duration and metabolic alterations by regulation on dietary intake. Therefore, exogenous dietary intake by appetite regulation is likely to be an important source of high cholesterol level in serum induced by sleep disturbance.

Regulation of hepatic cholesterol metabolism is controlled by the circadian clock (Pan et al., 2013; Ma et al., 2015). Oscillatory transcription of genes encoding key enzymes regulating cholesterol homoeostasis is tightly controlled during day and night. The results in this study showed that CYP7A1 expression was inhibited but with HMGCR expression unchanged induced by sleep disturbance. Therefore, conversion of cholesterol to bile acids rather than cholesterol synthesis was more susceptible to disturbed sleep stress. CYP7A1 expression exhibits a strong circadian rhythm, which is negatively regulated by peroxisome proliferator-activated receptor a, DEC1/2, and E4BP4, and positively regulated by NR1D1 and DBP (Noshiro et al., 2007; Duez et al., 2008). In this study, expressions NR1D1 were downregulated, and exhibit rapid response to SD at ZT4. Moreover, sleep disturbance could exacerbate the reduction of NR1D1 mediated CYP7A1 expression. Accordingly, the results provided a way to understand how circadian clocks link sleep disturbance to cholesterol metabolism by NR1D1. Additionally, the results partially explained the endogenous regulatory mechanism of liver cholesterol accumulation. However, NR1D1 acts as a transcriptional repressor in the inhibition of BMAL1 transcription and regulation of biological processes, such as adipocyte differentiation and lipid metabolism (Wang and Lazar, 2008; Le Martelot et al., 2009). Since both NR1D1 and CYP7A1 expression were downregulated in this study, regulation of CYP7A1 expression should be an indirect mechanism mediated by NR1D1 through negative regulators of CYP7A1 rather than positive regulators. The involvements of negative regulators of CYP7A1 in this study remains to be explored in our future work.

In summary, our study has demonstrated that an increased serum and liver cholesterol level induced by sleep disturbance is an early pathological event, which closely correlates with a decreased conversion of cholesterol to bile acids by NR1D1 mediated CYP7A1 inhibition. Thus, our finding explained the important role of the circadian clock in linking sleep loss and lipid profile abnormality.

## Data Availability Statement

The original contributions presented in the study are included in the article/Supplementary Material and further inquiries can be directed to the corresponding authors.

## Ethics Statement

The animal study was reviewed and approved by the Animal Ethics Committee of the Beijing Institute of Basic Medical Sciences.

## Author Contributions

XH and YZ were the co-first authors who performed most of the experiments and data analysis. WW, CZ, and LW performed the animal experiments. MD and MZ contributed to animal breeding. CX designed, performed, and supervised the study and wrote the paper. LS provided suggestions and guidance of this study and revised the paper. All authors contributed to the article and approved the submitted version.

### Conflict of Interest

The authors declare that the research was conducted in the absence of any commercial or financial relationships that could be construed as a potential conflict of interest.

## Supplementary Material

Supplementary Figure 1 CYP8B1 and CYP27A1 expression under sleep deprivation (SD) condition. **(A)** The protein expression of CYP8B1 and CYP27A1 in liver under SD condition. **(B)** Relative mRNA expression of CYP8B1 in livers after SD.

## Abbreviations

CVDs: Cardiovascular diseases
SD: Sleep deprivation
ARNTL: Aryl hydrocarbon receptor nuclear translocator-like
PER2: PERIOD2
CRY1/CRY2: Cryptochrom1/2
NR1D1: Nuclear receptor subfamily 1, group D, member 1
HDL: High density lipoprotein
VLDL: Very low density lipoprotein
8-OHdG: 8-Hydroxyguanosine
ALP: Alkaline phosphatase
HMGCR: 3-Hydroxy-3-methylglutaryl-CoA reductase
CYP7A1: Cytochrome P450 family 7 subfamily A member 1
FXR: Farnesoid-X-receptor
RXRa: Retinoid X receptor alpha
LRH1: Liver receptor homolog 1
HNF4a: Hepatocyte nuclear receptor 4 alpha
C/EBPa: CCAAT enhancer binding protein α
FOXO1: Forkhead box O1
E4BP4: E4, promoter binding protein 4
