# Supplementary figures and images for: Sleep Disturbance Induces Increased Cholesterol Level by NR1D1 Mediated CYP7A1 Inhibition
Source: Front Genet. 2020 Dec 23;11:610496. doi: 10.3389/fgene.2020.610496 (PMC7793681; doi:10.3389/fgene.2020.610496)

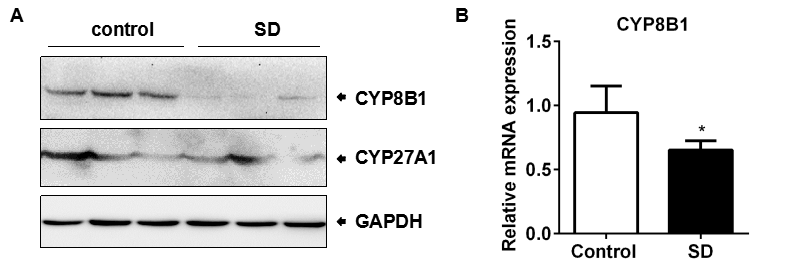

Supplement: Supplementary file 1 [file Image_1.tif]
